# A systematic review with meta-analyses of the association between stigma and chronic pain outcomes

**DOI:** 10.1097/j.pain.0000000000003243

**Published:** 2024-05-16

**Authors:** Lauren M. Hickling, Selsebil Allani, Matteo Cella, Whitney Scott

**Affiliations:** a King's College London, Department of Psychology, Institute of Psychiatry, Psychology, and Neuroscience, London, United Kingdom; b South London and Maudsley NHS Foundation Trust, London, United Kingdom; c INPUT Pain Management Unit, Guy's and St Thomas' NHS Foundation Trust, London, United Kingdom

**Keywords:** Chronic pain, Stigma, Systematic review, Meta-analysis

## Abstract

Stigma is common in people experiencing chronic pain and there are indications that it may adversely affect pain outcomes. However, to date, there is no systematic review exploring the impact of stigma on chronic pain-related outcomes. This systematic review and meta-analysis aimed to examine the association between stigma and key chronic pain outcomes and differences in stigma between pain conditions. Seven databases were searched for studies reporting a measure of association between stigma and at least one pain outcome in adults with chronic pain. Studies were screened by 2 independent researchers. Nineteen studies met eligibility criteria and data were extracted, quality-assessed, and narratively synthesised and meta-analysed where possible. Meta-analyses of bivariate cross-sectional correlations demonstrated significant positive correlations between stigma and pain intensity, disability, and depression, with small to moderate effects. Data from 2 prospective studies and those only reporting multivariate analyses that were not included in meta-analyses further supported these findings. There was some evidence that individuals who experience pain conditions with less clear pathophysiology may report greater stigma, although more research is needed. The review highlights that there is a growing number of studies on stigma in the pain field showing an adverse association between stigma and chronic pain outcomes.

## Abstract 2

Supplemental Digital Content is Available in the Text.

## 1. Introduction

Chronic pain is one of the leading causes of disability worldwide.^61^ The biopsychosocial model continues to be a key overarching framework for understanding chronic pain.^42^ Despite the relevance of social factors within this model, relatively less research has focussed on the social context of chronic pain.^27^

One of the many social factors that is increasingly recognised as relevant to chronic pain is the experience of stigma. Stigma was defined by Goffman^20^ as a phenomenon in which someone is discredited, considered less desirable, dangerous, or weak, because of an attribute or stereotype perceived by others. This definition was later adapted and expanded upon by Link and Phelan^32^ to include 4 related components: (1) people label differences between individuals; (2) cultural beliefs are used to link those with these labels to negative/undesirable stereotypes; (3) labelled individuals are separated into “us; and them” categories; and (4) those labelled experience loss of status and discrimination.

Emerging literature suggests stigma may be common among people experiencing chronic pain.^24,45,52^ Indeed, average stigma scores in a sample of people with chronic pain (n = 300) were more than one standard deviation higher than average scores for other long-term conditions, such as multiple sclerosis and Parkinson's.^39,52^ In 2016, De Ruddere and Craig^15^ published a nonsystematic review in which they describe potential mechanisms underlying stigmatising responses toward people in pain. These include a lack of explanation for pain, personal beliefs about pain/illness, and evolutionary influences such that pain may represent a threat of a communicable disease.^15,29^ Supporting these ideas, vignette studies suggest that observers report less sympathy and are less inclined to help when there is no clear biomedical evidence for pain.^16^ In addition, research shows that people with fibromyalgia, a complex condition with poorly understood pathophysiology, report greater pain invalidation compared with people with rheumatoid arthritis, which has clearer biomedical causes.^28^ Finally, mixed-methods research shows that pain is often disbelieved because of its invisible nature and can be viewed by others as an excuse for “laziness” or “drug-taking”.^2^

De Ruddere and Craig^15^ suggested that stigma from others may be internalised by the individual in pain and may contribute to poorer pain-related outcomes. Their review outlines a small but growing number of studies investigating the impact of stigma on people with pain, and how stigma may affect pain-related disability and distress.^15^ Since this topical review, there have been further studies reporting on the association between stigma and chronic pain outcomes. However, these have not been systematically reviewed and meta-analysed, which limits a comprehensive understanding of the state of this area of research. To address this gap, the aim of this review was to systematically identify, synthesise, and critically evaluate the existing evidence on the association between stigma and key chronic pain outcomes. This systematic review aimed to answer the following questions.

(1) Is stigma quantitatively associated with pain outcomes in people with chronic pain and how strong are these associations?
(2) Do levels of stigma differ across different pain conditions?

## 2. Methods

This review was conducted and reported in accordance with the “Preferred Reporting Items for Systematic Reviews and Meta-Analyses” (PRISMA) guidelines,^38^ and was pre-registered on PROSPERO (registration number: CRD42021283263).

### 2.1. Eligibility criteria

### 2.2. Inclusion

Studies were included in the review if they: (1) included participants older than 18 years; (2) recruited people with a chronic (≥3 months^26^) nonmalignant pain condition; (3) included a measure of stigma, as defined by the manuscript authors; (4) were available in full-text in English; and (5) used a cross-sectional, case-control, prospective, or randomised-controlled trial (RCT) design that reported a measure of association between stigma and at least one pain outcome (ie, the presence of pain, pain intensity, pain-related disability/functioning, depression, anxiety, or quality of life). Mixed-methods studies were included if they reported a quantitative measure of association between stigma and a pain outcome. Measures of stigma could be previously validated or developed for the study. Stigma measures could be general, pain-specific, or specific to another health condition, such as stigma related to the human immunodeficiency virus (HIV).

### 2.3. Exclusion

Studies were excluded if they (1) reported exclusively qualitative data; (2) were conference papers, book chapters, conference abstracts, or correspondence; (3) were theoretical or methodological papers; or (4) investigated samples with chronic pain secondary to cancer, acute pain (<3 months), postsurgical pain (or any other pain) of an unspecified duration, or studies investigating headache due to different presumed pathophysiological mechanisms. If a study measured stigma and one or more pain outcome in a sample with chronic pain but did not report on a measure of association between these, study authors were contacted to ask if they could provide the data; however, if the authors did not provide this within 4 weeks, the study was excluded. In addition, if it was unclear whether a sample was composed of people with chronic pain, then authors were contacted to clarify. If no response was given, or if the response could not confirm the presence of chronic pain, the study was excluded.

### 2.4. Information sources and search strategy

Five databases were searched for published literature: Medline, Embase (Ovid), CINAHL, PsycINFO (Ovid), and Web of Science. Grey literature was also searched using OpenGrey (accessed via Data Archiving and Networked Services EASY Archive). At the time of commencing the systematic search, the online database for OpenGrey had been shut down. A copy of the database was accessed at: https://easy.dans.knaw.nl/ui/datasets/id/easy-dataset:200362. The search was conducted on this archived database and Psyarxiv. Reference lists of previous systematic and scoping reviews and eligible full-text papers were searched. Databases were searched from inception to April 11, 2022. An updated search was conducted on August 21, 2023. The search terms covered terms related to “stigma” and “chronic pain” and were limited to studies involving adult human participants and published in English, where database searches allowed. The search terms used were based on previous reviews and meta-analyses on stigma and/or chronic pain^17, 50, 57, 66^ (supplemental digital content, Appendix A, http://links.lww.com/PAIN/C38).

### 2.5. Study selection

Duplicates were removed using Endnote 20 and then checked by the lead reviewer (LH). Titles and abstracts were independently screened by 2 reviewers (L.H. and S.A.). Agreement between the reviewers on titles and abstracts at the first search was 94.9% (Cohen's κ = 0.77) and 96.2% (Cohen's κ = 0.80) at the second search, indicating substantial agreement. Any disagreements were subsequently discussed with a third reviewer (W.S.) until consensus was reached. Eligible full-text papers were retrieved and screened using the same protocol.

### 2.6. Data extraction

Data for eligible studies were extracted into a Microsoft Excel spreadsheet designed for this review. Data extracted included study characteristics (setting, design, sample size, and participant demographics); exposure (stigma definition and measure); and pain-related outcomes and measures used. Bivariate correlations, regression coefficients, odds/risk ratios (OR/RR), and/or ANCOVAs reporting on the association between stigma and pain outcomes were extracted depending on what was reported. To facilitate comparison across studies and maximise the number eligible for meta-analyses, the bivariate correlation was prioritised for data extraction.^50^ Because covariates in multivariate analyses varied across studies, multivariate analyses were only included if no bivariate correlation was reported. Where available, means and standard deviations were extracted to compute Hedges' *g* for comparisons of stigma for people with and without chronic pain and to compare stigma levels across different pain conditions. Where more than one stigma measure was used, data for the measure most commonly used measure were extracted. If this was unclear from the literature, the longest measure was extracted.^66^

One study reported subgroups with different pain/health conditions.^34^ For this study, reviewers extracted the relevant measures of association within the fibromyalgia and rheumatoid arthritis subgroups. The data that were extracted did not always correspond to the overall study design. For example, in some cases, baseline cross-sectional correlation data were extracted from studies with a prospective design where stigma data were not reported longitudinally. In these cases, the design of the overall study was noted, but the narrative synthesis/meta-analysis reports that the data included are cross-sectional. Data extraction was completed independently by 2 reviewers and any discrepancies agreed with a third reviewer.

### 2.7. Quality assessment

Study quality was assessed using the Critical Appraisal Skills Programme (CASP) cohort study checklist.^10^ This was independently rated by 2 reviewers with any discrepancies resolved in discussion with a third. CASP items were rated as “yes,” “no,” or “somewhat/can't tell”, and these were then converted to a colour scheme where “green” is low risk of bias, “red” is high risk, and “amber” is moderate risk. This was used as the CASP checklist does not suggest an overall scoring system,^10^ and this has been a method that has been previously recommended.^3,5,48,55^ The CASP was used as it includes checklists covering a range of study designs that were eligible for this review. The tool covers 3 broad issues: study validity; precision and confidence in the results; and generalisability. Where cross-sectional data were extracted within a prospective design, only the cross-sectional items of the CASP were completed. Methods to assess confidence in the body of evidence for each outcome were not specified in the protocol. However, the robustness of the meta-analytic results is interpreted based on the relative number of studies/participants for each outcome.

### 2.8. Data synthesis

All studies reporting an association between stigma and pain-related outcomes were included in the narrative synthesis.^46^ Quantitative synthesis was conducted when 2 or more studies reported the same measure of effect (eg, *r*) between stigma and the same pain outcome and used the same design (eg, cross-sectional).^23^ For meta-analyses, *r* was transformed to Fisher's z to compute the pooled estimate.^12,53^

If a study separately reported the association of interest for different subscales of the stigma or pain outcome measure, then the average of the subscales was calculated and used. Some studies used measures of pain-related disability where higher scores indicate greater disability, while others used measures of functioning where higher scores indicate better functioning. For consistency in interpreting the direction of association, the sign for correlations using measures of functioning were reversed so that higher scores reflect higher pain-related disability.

Separate meta-analyses for each pain outcome were conducted using StataMP 17. Random-effects meta-analyses were conducted because heterogeneity across studies was expected.^4,44^ Statistical heterogeneity between studies was assessed using I^2^ and interpreted as low (<25%), medium (25%-50%), and high (>50%).^23^ Cohen's thresholds were adopted, whereby a correlation of *r* = 0.10 to 0.29 is small, *r* = 0.30 to 0.49 is moderate, and *r* ≥ 0.5 is large.^11^ For the between-groups comparisons, Hedges' *g* effects were interpreted as *g* < 0.20 is very small, *g* = 0.20 to 0.49 is small, *g* = 0.50 to 0.79 is moderate, and *g* ≥ 0.80 is large.^11^ No subgroup or sensitivity analyses were conducted, as per the protocol. Funnel plots were not appropriate to assess for publication bias, given the relatively small number of studies per analysis.

## 3. Results

**Figure 1.**
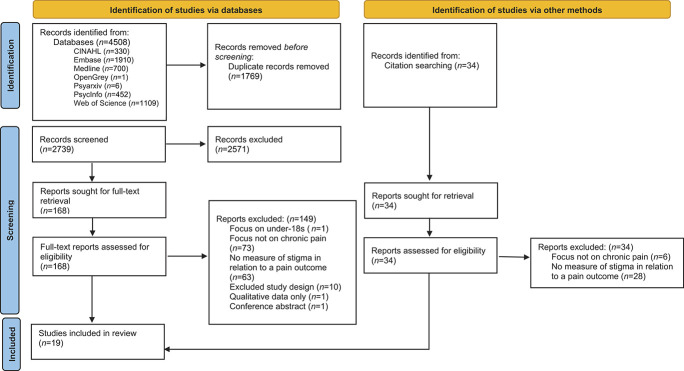
PRISMA flowchart based on Moher et al.^38^

The initial search resulted in a total of 3875 papers, after which 1508 duplicates were removed. After title and abstract and screening, 147 full texts were screened. After updating the search, there were a total of 4508 papers, after which 1769 duplicates were removed. After title and abstract screening, 168 full texts were screened (Fig. 1 for the PRISMA flowchart). Nineteen studies (18,822 participants; 7585 of these had chronic pain, 11,237 without pain for studies with a between-group design) were eligible for data extraction and inclusion in the narrative synthesis after full-text screening.

### 3.1. Study characteristics

The included studies were published between 1990 and 2023, with just under half (47.4%) conducted in the United States. The remaining studies were conducted in Canada, the United Kingdom, China, Belgium, South Africa, New Zealand, and Australia. Thirteen studies were cross-sectional,^2,21,22,24,33–35,40,41,47,49,63,64^ and one was prospective.^59^ There were 5 further studies that used prospective designs or that had prospective elements. One of these^45^ reported a prospective correlation between stigma and 2 of the pain outcomes, and a cross-sectional correlation between stigma and another pain outcome. The other 4 studies all reported on stigma in relation to chronic pain outcomes cross-sectionally.^36,52,58,62^

The studies considered samples with a range of chronic pain conditions. Studies of people with chronic pain and HIV^21,24,62,63^ and people with rheumatoid arthritis^22,33,34,59^ were the most common. Studies also included (sub)samples with fibromyalgia,^34,59^ burning mouth syndrome,^36^ interstitial cystitis,^49^ low back pain,^45^ temporomandibular pain,^35^ and vulvar pain.^41^ Six studies included people with pain conditions of mixed aetiology or did not specify pain-related diagnoses. The included studies had a wide range of sample sizes (median = 141, range = 16 - 12,384).

The mean participant age across studies ranged from 38.0 to 60.56 years.^35,36^ Across studies, most participants were female, with only 2 studies having more male participants.^21,24^ Four studies had an entirely female sample.^35,36,41,49^ Eleven studies provided information about the ethnic background of participants, with over half of these primarily recruiting White participants (*k* = 6). One study recruited only White participants,^35^ and 3 recruited samples comprised primarily of Black participants.^21,24,45^ The ethnicity of the participants in 2 further studies was undetermined, as they were described as being “Belgian”^59^ or “European”^2^ in origin.

Thirteen studies reported on the mean pain duration in the sample, which averaged 11.72 years (range: 6-19 years) across the studies. Six studies asked participants to report medication use for pain management,^2,21,24,35,45,49^ with around two-thirds taking medication for their pain (prescribed or unprescribed). Seven studies reported the comorbidities of participants.^2,21,24,40,41,49,59^ Three of these reported that the comorbidities were psychiatric in nature, with Naushad et al.^40^ reporting that 23.7% of their sample had diagnoses of chronic pain and major depression. Similarly, Goodin et al.^21^ noted that 75% of participants had a comorbid psychiatric diagnosis (36% of these were diagnosed with depression). Bean et al.^2^ specified that 49% of participants had a diagnosis of depression, 56% had an anxiety disorder, 26% had a diagnosis of post-traumatic stress disorder, and 9% were diagnosed with another mental health condition. Only 28% of participants were not diagnosed with a mental health condition. Hobson et al.^24^ provided no information as to the nature of participants' comorbidities, but noted they had a median of 4 other diagnoses. The remaining studies reported comorbidities of other pain diagnoses. A detailed summary of study characteristics can be found in Table 1.

**Table 1:** Study characteristics and definitions of stigma used.

Study (Year)	Country	Design	*N*	Type of pain	Measure of stigma	Definition of stigma
Mathur et al. 2023^36^	United States	Prospective*	16	Burning mouth syndrome	ISCP	“Stigma refers to convergence of cultural labels, stereotypes, discrimination, and social oppression that leads to unjust distribution of experiences and opportunities, and it is increasingly recognized as a social determinant of health disparities.” p.1213
Penn et al. 2020^45^	United States	Prospective†	105	Nonspecific chronic lower back pain	ISCP	“Disapproval/discrediting of, or discrimination against, a person who is deemed to possess undesirable characteristics that deviate from social norms” p.3162
Scott et al. 2019^52^	United Kingdom	Prospective*	293	Mixed	SSCI-8	“Devaluing and discrediting responses toward a person or group perceived to possess a negative attribute that deviates from social norms and involves elements of social exclusion and embarrassment” p.1165
Vallabh et al. 2014^58^	Canada	Prospective*	71	Unspecified	CPSS	Study authors refer to using the definition of Goffman^20^
Van Alboom et al. 2014^59^	Belgium	Prospective	198	Fibromyalgia and rheumatoid arthritis	Two daily assessment items adapted from the 3†I and 1 item from the SSCI	“Devaluing and discrediting responses of observers toward individuals who possess a characteristic that deviates from societal norms” p.350
Wadley et al. 2022^62^	South Africa	Prospective*	109	Chronic pain among people living with HIV	HSS	Unspecified
Bean et al. 2022^2^	New Zealand	Cross-sectional	215	Unspecified	ISCP	“Stereotypes or negative views attributed to a person or groups of people when their characteristics or behaviors are viewed as different from or inferior to societal norms” p.1749
Goodin et al. 2018^21^	United States	Cross-sectional	60	Chronic pain among people living with HIV	ISCP and HIV stigma mechanisms measure	“Devalued, blamed, and even report being dismissed by healthcare providers…can also become internalized, such that those with chronic pain report feeling inferior to others who do not have chronic pain” p.67
Han et al. 2023^22^	China	Cross-sectional	200	Rheumatoid arthritis	ISMI (Chinese version)	Study authors refer to using the definition of Link and Phelan^32^
Hobson et al. 2022^24^	United States	Cross-sectional	82	Chronic pain among people living with HIV	ISCP and HIV stigma mechanisms measure	“A type of social rejection that can produce “social pain” on behalf of the individual who experiences and internalizes the stigma” p.1
Liu et al. 2023^33^	China	Cross-sectional	141	Rheumatoid arthritis	SSCI-8	“Stigma refers to inner shame causing [sic] by the illness, which can be described as comprising enacted and internalized stigma” p.909
Looper and Kirmayer 2004^34^	Canada	Cross-sectional	74	Fibromyalgia and rheumatoid arthritis	Attitudes of others scale (items adapted from Explanatory Model Interview Catalogue and Pain Stigma Scale)	Unspecified
Marbach et al. 1990^35^	United States	Cross-sectional	151	Temporomandibular pain and dysfunction syndrome	SFPQ	“A complex process of social interaction between an unmarked or “normal" person and the bearer of an evident or presumed “mark" that defines the person as flawed, incomplete, spoiled, or undesirable” p.584
Naushad et al. 2018^40^	United States	Cross-sectional	236 (no pain: 121; chronic pain: 115)	Unspecified	PSSS and DSSS	Study authors refer to using the definitions of Goffman,^20^ and Link and Phelan^32^
Nguyen et al. 2013^41^	United States	Cross-sectional	12,834 (no pain: 7847; chronic pain: 4987)	Vulval pain	Two Likert scale questions based on the CPSS	Unspecified
Prunty et al. 2023^47^	United States	Cross-sectional	3821 (no pain: 3269; chronic pain: 552)	Unspecified	WBIS	“The devaluation of an individual or group due to weight or body size” p.33
Rabin et al. 2001^49^	United States	Cross-sectional	74	Interstitial cystitis	SSS	Unspecified
Wadley et al. 2019^63^	South Africa	Cross-sectional	50	Chronic pain among people living with HIV	HASI-P	“Acts perceived as stigmatising such as social exclusion or violence, or internalised stigma, whereby prevalent negative attitudes surrounding HIV are internalised and deemed valid by people living with HIV” p.2071
Waugh et al. 2014^64^	Australia	Cross-sectional	92	Mixed	ISCP (adapted from the ISMI)	“A subjective process, embedded within a socio-cultural context, which may be characterised by negative feelings (about self), maladaptive behaviour, identity transformation, or stereotype endorsement resulting from an individual's experience, perceptions, or anticipation of negative social reactions on the basis of their [condition]” p.550.e1

* Overall study (or elements of the study) prospective, but only reported cross-sectional association between stigma and pain outcomes so considered as cross-sectional data for the narrative synthesis/meta-analyses.

† Correlations between stigma and pain intensity and disability are prospective, while the correlation between stigma and depression was based on cross-sectional data.

3*I, Illness Invalidation Inventory; CPSS, Chronic Pain Stigma Scale; DSSS, Depression Self-Stigma Scale; HASI-P, HIV/AIDS Stigma Instrument-PWLA; HSS, HIV Stigma Scale; ISCP, Internalized Stigma of Chronic Pain Scale; ISMI, Internalized Stigma of Mental Illness Scale; PSSS, Pain Self-Stigma Scale; SFPQ, Stigma of Facial Pain Questionnaire; SSCI-8, Stigma Scale for Chronic Illnesses (Eight item version); SSS, Self-Stigmatization Scale; WBIS, Weight Bias Internalization Scale.

### 3.2. Definition and measurement of stigma

Four studies did not provide a definition of stigma although this was measured.^34,41,49,62^ The remainder adopted different definitions, including those of Goffman^20^ and Link and Phelan,^32^ and definitions that were developed by the study authors. Fourteen different tools were adopted to measure stigma, with the Internalised Stigma in People Living with Chronic Pain Scale (ISCP)^2,21,24,36,45,64^ being the most common. The eight-item Stigma Scale for Chronic Illnesses (SSCI-8),^33,52^ the Chronic Pain Stigma Scale (CPSS),^41,58^ and the HIV Stigma Mechanisms Scale (HIV-SMS)^21,24^ were each used in 2 studies. Almost half (*k* = 9) of the studies used a pain-specific stigma measure. Two studies used a general measure of stigma and a disease-/syndrome-specific measure. In 2 studies, chronic pain stigma and HIV stigma were assessed and combined to produce a measure of intersectional stigma.^21,24^ The remaining study measured pain stigma and depression stigma.^40^ Appendix B (supplemental digital content, http://links.lww.com/PAIN/C38) summarizes the assessment of pain outcomes across the studies.

### 3.3. Stigma and the presence vs absence of pain

Table 2 provides a synthesis of the findings of the associations between stigma and pain outcomes. Three cross-sectional studies examined the association between stigma and chronic pain status; Hedges' *g* for the between-groups comparison could not be computed. ANCOVA results from the study by Naushad et al.^40^ indicated that total stigma scores did not differ between people with chronic pain and people without chronic pain or depression (*P* = 0.81); however, participants with comorbid depression and chronic pain reported greater stigma than people without chronic pain or depression (*P* = 0.001). Nguyen et al.^41^ found that women with any kind of pain were more likely than women without pain to agree that doctors (adjusted relative risk [RR] = 1.44, 95% confidence interval [CI]: 1.36 to 1.52) and other people (adjusted RR = 1.58, 95% CI: 1.46-1.70) stigmatize pain. Finally, Prunty et al.^47^ found that weight self-stigma (OR = 1.50, 95% CI: 1.20-1.87, *P* < 0.001) was associated with increased odds of having chronic pain unrelated to arthritis (with chronic pain, n = 552; without chronic pain, n = 3269) vs not having chronic pain.

**Table 2:** Summary of the evidence for the association between stigma and pain outcomes.

Outcome	Bivariate findings	Multivariate findings
Presence vs absence of pain	N/A	Cross-sectional (*k* = 3): two studies^40,41^ showed significantly greater stigma in people with vs without pain (pain and comorbid depression analysis only for^40^). One study found that stigma was associated with increased odds of having chronic pain.^47^
Pain intensity	Prospective (*k* = 1): significant positive association between baseline stigma and follow-up pain (medium effect).^45^ Cross-sectional (*k* = 9): significant positive association (small effect) in meta-analysis (Fig. 2).	Cross-sectional (*k* = 1): increased pain intensity was significantly associated with the classes of moderate and high stigma relative to the low stigma class.^22^
Pain disability	Prospective (*k* = 1): significant positive correlation between baseline stigma and follow-up disability (medium effect).^45^ Cross-sectional (*k* = 6, 7 unique samples): significant positive association (medium effect) in meta-analysis (Fig. 3).	Prospective (*k* = 1): stigma significantly associated with pain-related disability within and between individuals.^59^
Depression	Cross-sectional (*k* = 10, 11 unique samples): significant positive association (medium effect) in meta-analysis (Fig. 4).	Cross-sectional (*k* = 3): significant association between stigma and depression across the studies.
Anxiety	Cross-sectional (*k* = 2): nonsignificant positive association (small effect) in meta-analysis (Fig. 5).	N/A
Quality of life	Cross-sectional (*k* = 1): significant negative association (medium effect).^58^	N/A
Stigma between pain conditions	Cross-sectional (*k* = 1): no significant difference in stigma between participants with fibromyalgia and RA.^34^	Prospective (*k* = 1): daily stigma not significantly different between fibromyalgia and RA, but daily stigma higher in comorbid fibromyalgia and RA group vs RA alone (small effect).^59^ Cross-sectional (*k* = 1): syndromic + nonsyndromic + vulvar pain reported significantly greater stigma vs nonsyndromic only.^41^

Multivariate findings only included in narrative synthesis when the study did not report bivariate measure of association.

RA, rheumatoid arthritis.

### 3.4. Stigma and pain intensity

**Figure 2.**
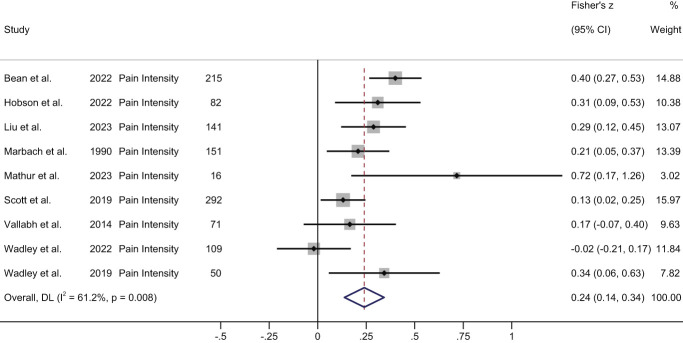
Forest plot of cross-sectional correlations between stigma and pain intensity. The grey boxes reflect the study weighting; the black diamonds indicate the effect for each study; the horizontal black lines show the 95% confidence intervals (CI); the red dotted line captures the pooled effect of all studies; the blue diamond shows the 95% CI of the pooled effect. Data from Wadley et al.^62^ were not reported in the published paper and were obtained following author contact.

Eleven studies (one prospective and 10 cross-sectional) reported on the association between stigma and pain intensity (Table 2). Penn et al.^45^ reported a significant correlation (medium effect) between baseline stigma and pain intensity reported one week later (n = 105, *r* = 0.41, *P* < 0.001). The meta-analysis of cross-sectional data from 9 studies demonstrated a small but significant pooled positive correlation (Fisher's Z = 0.24, 95% CI: 0.14-0.34, z = 4.59, *P* < 0.001; high heterogeneity, I^2^ = 61.2%) (Fig. 2). One further cross-sectional study (n = 200) examined multivariate predictors of latent classes of stigma.^22^ This study found that increased pain intensity was significantly associated with the classes of moderate stigma (OR = 1.54, 95% CI: 1.14-2.08, *P* = 0.005) and high stigma (OR = 1.80, 95% CI: 1.30-2.48, *P* < 0.001) relative to the low-stigma class.^22^

### 3.5. Pain-related disability

Eight studies (2 prospective and 6 cross-sectional) reported an association between stigma and pain-related disability (Table 2). In a prospective daily diary study (n = 198), Van Alboom et al.^59^ found that stigma was significantly associated with pain-related disability within (*B* = 0.07 [0.03], *P* < 0.05) and between (*B* = 0.15 [0.06], *P* < 0.05) individuals, controlling for covariates. Penn et al.^45^ reported a significant correlation (medium effect) between baseline stigma and pain-related disability 1 week later (n = 105, *r* = 0.39, *P* < 0.001).

**Figure 3.**
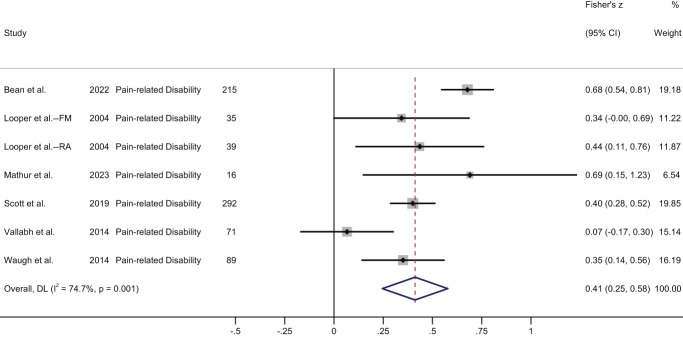
Forest plot of cross-sectional correlations between stigma and pain-related disability. FM, fibromyalgia; RA, rheumatoid arthritis.

Six studies reported cross-sectional bivariate correlations and were meta-analysed. Looper and Kirmayer^34^ reported correlations between stigma and disability separately for subgroups with fibromyalgia and rheumatoid arthritis, so these were included as separate samples. Also, one of the correlations reported in this study was written as *r* = 3.3; author contact clarified that this was an error and should be reported as *r* = 0.33. The meta-analysis of cross-sectional data demonstrated a moderate and significant pooled correlation (Fisher's Z = 0.41, 95% CI: 0.25-0.58, z = 4.86, *P* < 0.001; high heterogeneity, I^2^ = 74.7%). (Fig. 3).

### 3.6. Depression

**Figure 4.**
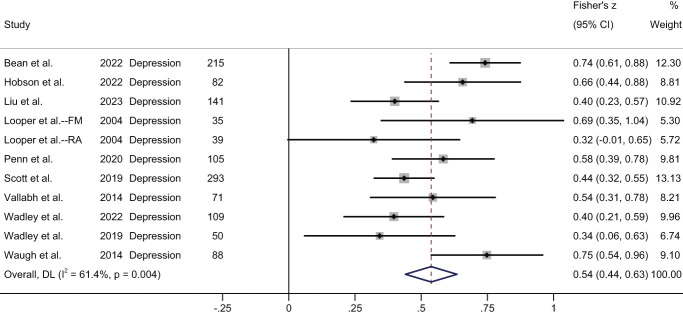
Forest plot of cross-sectional correlations between stigma and depression. FM, fibromyalgia; RA, rheumatoid arthritis. Data from Wadley et al.^62^ were not reported in the published paper and were obtained following author contact.

A measure of association between stigma and depression was reported in 13 studies with cross-sectional data, 10 of which included data for meta-analysis (Table 2). The fibromyalgia and rheumatoid arthritis subgroups from the study by Looper and Kirmayer^34^ were included as separate samples. The meta-analysis of cross-sectional data demonstrated a moderate significant pooled positive correlation (Fisher's Z = 0.54, 95% CI: 0.44-0.63, z = 10.82, *P* < 0.001; high heterogeneity, I^2^ = 61.4%) (Fig. 4).

Three cross-sectional studies reported multivariate analyses only. After controlling for covariates, stigma was significantly associated with depression in the studies by Goodin et al.^21^ (F [2, 51] = 4.07, *P* = 0.02) and Rabin et al.^49^ (n = 74, B = 0.20, independent variance = 0.06, *P* < 0.05). Also, after controlling for covariates, Naushad et al.^40^ found that total stigma scores were significantly higher in the group with comorbid pain and depression compared with the group with chronic pain only (F [1, 107] = 9.07, partial η^2^ = 0.08, *P* = 0.003).

### 3.7. Anxiety

**Figure 5.**
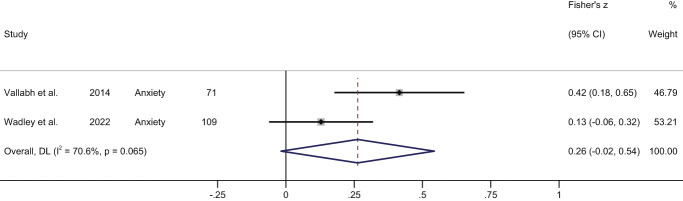
Forest plot of cross-sectional correlations between stigma and anxiety. Data from Wadley et al.^62^ were not reported in the published paper and were obtained following author contact.

Only 2 studies reported a measure of association between stigma and anxiety (Table 2). The meta-analysis of cross-sectional data demonstrated a small but nonsignificant pooled correlation (Fisher's Z = 0.26, 95% CI: −0.02 to 0.54, z = 1.84, *P* = 0.07; high heterogeneity, I^2^ = 70.6%) (Fig. 5).

### 3.8. Quality of life

Only one cross-sectional study reported a measure of association between stigma and quality of life (Table 2). Vallabh et al.^58^ (n = 71) reported a significant negative correlation between stigma and quality of life (*r* = -0.47, *P* = 0.001; medium effect).

### 3.9. Levels of stigma between pain conditions

One prospective daily diary study found that daily stigma levels were not significantly different between participants with fibromyalgia and rheumatoid arthritis (*P* = 0.06; *g* = 0.21, small effect), or those with both conditions compared with those with fibromyalgia only (*P* = 0.32; *g* = 0.16, less than small effect).^59^ However, stigma was higher in those with comorbid fibromyalgia and rheumatoid arthritis compared with those with rheumatoid arthritis alone (*P* = 0.02; *g* = 0.39, small effect).^59^

Two cross-sectional studies compared stigma between chronic pain conditions. In the first, Looper and Kirmayer^34^ found no significant differences in stigma between participants with fibromyalgia and rheumatoid arthritis (*P* = 0.10; *g* = 0.39, small effect). Nguyen et al.^41^ examined 2 stigma items among women with various combinations of “syndromic” (eg, interstitial cystitis and fibromyalgia), “nonsyndromic” (eg, endometriosis), and vulvar pain conditions relative to women with nonsyndromic pain only. Women with all 3 kinds of pain were mostly likely to agree that doctors stigmatize pain compared with women with nonsyndromic pain only (adjusted RR = 1.69, 95% CI: 1.46-1.97). By contrast, women with vulvar pain and syndromic pain were most likely to agree that other people stigmatize pain compared with women with nonsyndromic pain only (adjusted RR = 2.35, 95% CI: 1.83-3.02). Comparisons between the other combinations of the pain conditions were not conducted in this study.^41^

### 3.10. Quality assessment

Most studies were rated positively for most of the CASP items. Although some studies used unvalidated measures, the studies that did report Cronbach's alpha had moderate to high internal consistencies. Stigma and pain outcomes were assessed using self-report, which is appropriate, given their subjective nature. The most common limitation was a lack of or unclear precision in the results (CASP item 7). For studies included in our meta-analyses, precision was judged based on the width of the confidence intervals from these analyses. Some studies not included in the meta-analyses did not report confidence intervals and we were therefore unable to determine the precision of results. (CASP item 7). Relatedly, a number of studies had relatively small samples, which limits confidence in the results (CASP item 8). There were also several studies that did not sufficiently consider or account for potential confounding variables (CASP items 5a and 5b). A detailed summary of the quality assessment findings can be found in Table 3. Items 6a and 6b pertained to the follow-up period of prospective studies and are thus not applicable for cross-sectional studies/data. One prospective study had a 100% retention rate for a 1-week follow-up,^45^ while the other reported 90% completion of daily diary assessments over a 2-week period.^59^

**Table 3:** Quality appraisal.

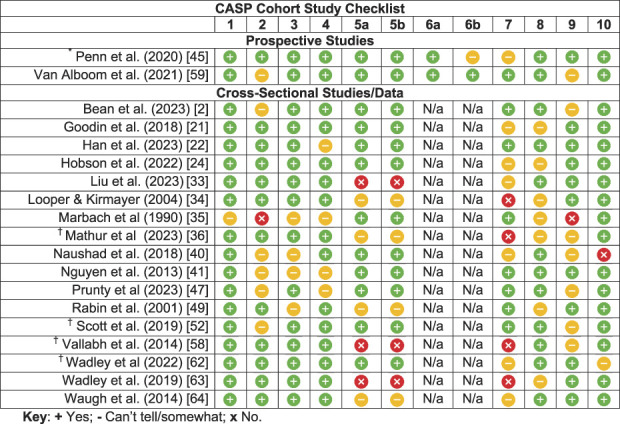

Key indicates: +, yes; −, can't tell/somewhat; x; no.

* Correlations between stigma and pain intensity and disability were prospective, while the correlation between stigma and depression was based on cross-sectional data.

† Overall study (or elements of the study) prospective, but only reported cross-sectional association between stigma and pain outcomes so considered as cross-sectional data.

N/a, prospective data were not extracted from this study and therefore only the cross-sectional quality items were applied.

Given the small number of studies and participants for the syntheses for anxiety and quality of life, there is reduced confidence in the reliability of these results. The syntheses and meta-analyses for pain intensity, disability, and depression are based on a relatively larger number of studies/participants and are therefore more likely to be reliable.

## 4. Discussion

This review systematically investigated whether stigma is associated with key chronic pain–related outcomes. Meta-analyses of cross-sectional studies demonstrated significant positive correlations between stigma and pain intensity, disability, and depression. Prospective data from 2 studies and studies only reporting multivariate analyses further support these findings. The associations between stigma and anxiety and quality of life must be interpreted cautiously as they come from very few studies with small samples. Taken together, the results suggest that stigma is important to address to improve outcomes among people with chronic pain and should be considered in future high-quality research.

This review highlights that a range of definitions of stigma have been used. Some of these are clear, well defined, and commonly used (such as those by Goffman^20^ and Link and Phelan^32^), whereas some studies do not provide a clear definition. Relatedly, studies adopted a range of stigma measures. Notably, the pattern of findings was generally consistent across the measures and definitions used. Given the potential for pain-related stigma to intersect with the stigma of other comorbid physical or mental health conditions, future research may carefully consider whether using more than one stigma measure is useful. Indeed, the studies by Goodin et al.^21^ and Hobson et al.^24^ in this review found that people living with HIV and chronic pain who reported high levels of HIV and pain stigma experienced the greatest severity of pain intensity, depression symptoms, and insomnia.

Results suggest that the association between stigma and depression may be stronger than the associations between stigma and pain intensity and disability. Research is needed to understand factors that explain the stronger association between stigma and depression. Of course, given that most studies were cross-sectional, the direction of this relationship is difficult to disentangle. It is also plausible that people with pain and more severe depression experience increased stigma, given the stigma associated with mental illness.^18^ However, one prospective study in people living with HIV that was excluded from this review because of focusing on acute pain provides initial support for potential directions of effect.^13^ That study found that baseline internalised HIV stigma predicted increased acute pain 1 year later, and this was mediated by depressive symptoms at 6 months.^13^ Longitudinal research is needed to investigate whether this pattern of results replicates in people with persistent pain.

Only a few studies have compared levels of stigma between people with and without pain and between different pain conditions. The current review provides some evidence that, unsurprisingly, people with pain experience greater stigma than people without pain, and this may be explained by comorbid mental illness.^40^ This again highlights the need to examine how pain and mental health stigma intersect. The review provides some evidence that individuals who experience pain conditions with less clear pathophysiology report greater stigma. However, differences in the pain conditions and categories examined across studies make it difficult to interpret these findings. Further research is needed to build on these findings and, importantly, to understand factors that exacerbate stigma across conditions, which may ultimately inform stigma reduction interventions.

During screening, we identified several studies examining stigma in adults with sickle cell disease, which were excluded as we could not determine whether the samples had acute vs chronic pain as per our inclusion criterion.^1,6,25,43^ Nonetheless, stigma is clearly relevant for people with sickle cell disease.^7^ A previous systematic review and more recent studies indicate that stigma in people with sickle cell disease is associated with poorer mental health, delayed emergency department treatment for acute pain crises, and increased pain interference.^1,6,7,25,43^ Given that sickle cell disease predominantly affects individuals from African and Caribbean backgrounds, it is important to investigate how racism contributes to stigma in this population.^7^

This review identified several other psychosocial constructs that were measured alongside stigma in the included studies. Some of these other constructs reflect variables within widely adopted psychological models of pain, namely the fear-avoidance^14,30,60^ and psychological flexibility models.^37^ Importantly, a key limitation of these models is that they fail to adequately acknowledge social factors such as stigma. Based on the current review's findings, further development of theory is needed to understand the role of stigma in chronic pain, including how stigma relates to well-studied psychological constructs in the pain field and other closely related constructs, such as discrimination,^51^ invalidation,^28^ and injustice experiences.^9^ Developments within the fear-avoidance and psychological flexibility models to further specify how stigma interacts with psychological processes within these models to impact on pain outcomes may be useful.

To further develop theory in this area, it will also be helpful to draw on models of stigma from other fields. For example, Stangl et al.^54^ outline a framework for stigma and discrimination across a range of health conditions. Their framework describes how stigma occurs across the socioecological spectrum, reflecting individual, interpersonal, organisational, community, and public policy contexts.^54^ Across these levels, there are drivers and facilitators of stigma, which may include cultural norms/beliefs (eg, about the nature of pain) and political and economic narratives around “productivity,”^19,54^ for example. These drivers and facilitators influence stigma “marking” where stigma is applied to people with a particular health condition (ie, chronic pain). People with pain may be “marked” with multiple intersecting stigmas related to comorbidities (eg, mental illness and HIV) and identity-related factors, such as “race” and gender.^54^ This marking then “manifests” in the lived experience of stigma, which affects outcomes (eg, lack of appropriate treatments, unfair employment practices, etc.) and, ultimately, health, quality of life, and social inclusion.^54^ Research is needed to understand each of these domains across different levels of the system in the context of chronic pain.

The current findings highlight the need for research on strategies to reduce stigma and its impact on people with pain. Although not the focus of this review, 2 studies did report on changes in stigma during acceptance and commitment therapy (ACT)^52^ and cognitive-behavioural therapy (CBT).^58^ Scott et al.^52^ found that total stigma scores on a measure of enacted and internalized stigma did not improve during an intensive pain management programme based on ACT; however, a small but significant reduction on internalised stigma was observed. ACT does not aim to directly alter experiences such as thoughts and feelings related to stigma, but rather it focuses on improving psychological flexibility in response to these difficult experiences.^37^ As such, future research might benefit from a more fine-grained analysis of the relationship between stigma and psychological flexibility after treatment. Consistent with the psychological flexibility model,^37^ it may be that increases in psychological flexibility after ACT buffer the impact of stigma on functioning and mental health. Interestingly, Vallabh et al.^58^ found that a CBT-based family intervention significantly decreased the level of perceived stigma during interactions with physicians; however, there was no significant change in stigma perceived during interactions with the general public or family. Therefore, further interventional research is needed to understand how best to address pain-related stigma.

Because of the complexity of the stigma experience, it is necessary to address it at multiple levels, such as the institutional and group levels, rather than just at an individual level.^52,65^ Possible strategies may include adapting policies to make them more inclusive^27,65^ and increasing empathy and validation for people with pain.^8^ Drawing on work from other fields, for example, from 2007 to 2021, the “Time to Change”^56^ campaign aimed to reduce stigma and discrimination around mental health, working with employers, schools, and communities to educate people about mental health and empower those with mental health conditions to speak out against stigma. Although this is an ongoing project, research by the campaign leaders showed an improvement in attitudes towards mental health.^56^ However, more research is needed to understand how best to target chronic pain stigma at different levels of the social system.

Several limitations of the primary studies are notable. As mentioned, the studies were mostly cross-sectional, limiting conclusions about the direction of relationships between variables. Therefore, future research should focus on prospective designs and examine the contribution that stigma makes to key pain outcomes over time when controlling for baseline scores on those outcomes (and vice versa). In addition, with a few exceptions, the studies generally had relatively small samples. This limits the precision of the estimates and may risk false positives or underpowered analyses. Another limitation was the heterogeneity and sometimes absence of a definition for stigma. Clearly defining stigma is of utmost importance, and researchers should ensure that assessment measures appropriately correspond to the chosen definition and are well validated and psychometrically sound. Greater consistency in the definition and measurement of stigma across studies will enable understanding of the reliability and generalizability of effects.

Several limitations of the review must also be considered. Although there was sufficient bivariate data for meta-analyses, some pooled correlations had more contributing evidence/studies than others and there were instances of missing data that could not be rectified by contacting study authors. Therefore, interpretation of the findings should consider the potential bias caused by missing data. Studies were assessed using a quality assessment tool that does not have scoring benchmarks or cutoffs, which limits statements that can be made about the overall quality of individual studies. It is also possible that the decision to include only research published in English restricted the research included and may perpetuate inequities with respect to increasing diversity within research samples.^31^ Future research should focus on studies with more diverse samples. It should be noted that although studies were assessed in terms of whether they controlled for confounding variables, the present review focused primarily on bivariate associations to facilitate meta-analyses. Where reported in the narrative synthesis, the multivariate findings were largely consistent with the bivariate analyses, but this limits comprehensive understanding of the unique role of stigma relative to other variables.

Despite these limitations, this systematic review provides evidence of associations between stigma and chronic pain–related outcomes. The review highlights the importance of addressing stigma to improve the lives of people with chronic pain. Further research is needed to understand the factors that contribute to stigma among people with chronic pain, including how pain-related stigma intersects with the stigma of other comorbid conditions and aspects of a person's social identity. Theoretically informed research is also needed to understand how to optimally intervene at different levels to target stigma and its adverse impacts on people with chronic pain.

## Conflict of interest statement

The authors have no conflicts of interest to declare.

## Appendix A. Supplemental digital content

Supplemental digital content associated with this article can be found online at http://links.lww.com/PAIN/C38.

## Supplemental video content

A video abstract associated with this article can be found on the *PAIN* website.

## Supplementary Material

SUPPLEMENTARY MATERIAL
