# Supplementary material for: A systematic review with meta-analyses of the association between stigma and chronic pain outcomes
Source: Pain. 2024 May 16;165(8):1689–701. doi: 10.1097/j.pain.0000000000003243 (PMC11247453; doi:10.1097/j.pain.0000000000003243)
Supplement: SUPPLEMENTARY MATERIAL [file jop-165-1689-s001.pdf]

Appendix A: Systematic Review Search Terms

				Grey Literature	
**Medline / EMBASE (OVID)**	**Web of Science**	**CINAHL**	**PsychInfo (OVID)**	**OpenGrey**	**Psyartix**
1. exp PAIN/	1. ALL=(pain)	1. MH PAIN	S1. exp PAIN/	Stigma AND Pain	Stigma AND Pain
2. pain.ti,ab	2. AB=(chronic NEAR/2 pain*)	2. AB pain	S2. pain.ti,ab		
3. (chronic adj2 pain*).tw.	3. AB=(chronic NEAR/2 (discomfort or ach* or neuralgi* or dysmenorrhea))	3. AB chronic N2 pain*	S3. (chronic adj2 pain*).tw.		
4. (chronic adj2 (discomfort or ach* or neuralgi* or dysmenorrhea)).tw.	4. AB=fibromyalgi*	4. AB chronic N2 (discomfort or ach* or neuralgi* or dysmenorrhea)	S4. (chronic adj2 (discomfort or ach* or neuralgi* or dysmenorrhea)).tw.		
5. exp fibromyalgia/	5. AB=neuropath* NEAR/2 pain	5. MH "Fibromyalgia+"	S5. “Fibromyalgia”.mp.		
6.fibromyalgia*.tw.	6. ALL=neuropath*	6. AB fibromyalgi*	S6. neuropath* adj2 pain.mp.		
7.neuropath* adj2 pain.mp.	7. AB=(chronic* NEAR/5 neuralgi*)	7. AB neuropath* N2 pain	S7. neuropath*.mp.		
8.neuropath*	8. AB=(chronic* NEAR/5 arthralgi*)	8. AB neuropath*	S8. (chronic* adj5 neuralgia).tw.		
9.(chronic* adj5 neuralgi*).tw.	9. ((((((#1) or #2) or #3) or #4) or #5) or #6) or #7) or #8	9. AB chronic* N5 neuralgi*	S9. (chronic* adj5 arthralgia).tw.		
10. (chronic* adj5 arthralgi*).tw.	10. AB=stigma	10. AB chronic* N5 arthralgi*	S10. S1 OR S2 OR S3 OR S4 OR S5 OR S6 OR S7 OR S8 OR S9		
11. 1 OR 2 OR 3 OR 4 OR 5 OR 6 OR 7 OR 8 OR 9 OR 10	11. ALL=Self?stigma*	11. 1 OR 2 OR 3 OR 4 OR 5 OR 6 OR 7 OR 8 OR 9 OR 10	S11. exp SOCIAL STIGMA/		
12. exp SOCIAL STIGMA/	12. ALL=internali?ed stigma	12. (MH "Stigma+")	S12. exp SOCIAL REJECTION/		
13. exp SOCIAL REJECTION	13. ((#10) or #11) or #12	13. AB stigma	S13. stigma*.mp.		
14. stigma.mp.	14. (#9) and 13	14. AB self?stigma*	S14. self?stigma*.mp.		
15. self?stigma*.mp.		15. AB internali?ed stigma	S15. internali?ed stigma.mp.		
16. internali?ed stigma.mp.		16. 12 OR 13 OR 14 OR 15	S16. S11 OR S12 OR S13 OR S14 OR S15		
17. 12 OR 13 OR 14 OR 15 OR 16		17. 11 AND 16	S17. S10 AND S16		
18. 11 AND 17					

Appendix B: Measurement of pain outcomes across studies

	Presence vs absence	Pain intensity	Disability	Depression	Anxiety	Quality of life	Stigma between pain conditions
Bean et al. (2023) [2]		BPI	BPI	PHQ-9			
Goodin et al. (2018) [21]				CES-D			
Han et al. (2023) [22]		VAS					
Hobson et al. (2022) [24]		BPI		CES-D			
Liu et al. (2023) [33]		VAS		ADDI-27			
Looper & Kirmayer (2004) [34]			SF-36	SCL-90-R			Diagnosis of fibromyalgia and RA
Marbach et al. (1990) [35]		Composite Pain Severity Score					
Mathur et al. (2023) [36]		BPI	BPI				
Naushad et al. (2018) [40]	Self-reported chronic pain			SCID			
Nguyen et al. (2013) [41]	Self-reported chronic pain						Self-reported chronic pain classified into seven categories
Penn et al. (2020) [45] †		SPPB	SPPB	CES-D			
Prunty et al. (2023) [47]	Self-reported chronic pain						
Rabin et al. (2001) [49]				CES-D			
Scott et al. (2019) [52] †		VAS	BPI				

Vallabh et al. (2014) [58]†		MPQ	PDI	HAD-II	HAD-II	15-D	
Van Alboom et al. (2021) [59]*			GCPS				Diagnosis of fibromyalgia or RA
Wadley et al. 2022 [62] †		BPI		PHQ-9	HSC-25		
Wadley et al. (2019) [63]		VAS		BDI-II			
Waugh et al. (2014) [64]			RMDQ	DASS-21			

Abbreviations: **ADDI-27,** Anxiety Depression Distress Inventory-27 items; **BDI**, Beck Depression Inventory; **BPI**, Brief Pain Inventory; **CES-D**, Center for Epidemiological Studies Depression Scale; **DASS**, Depression Anxiety and Stress Scale; **EDSS**, Expanded Disability Status Scale; **GCPS**, Graded Chronic Pain Scale; **HAD**, Hospital Anxiety & Depression; **HSC**, Hopkins Symptom Checklist; **MPQ**, McGill Pain Questionnaire; **PDI**, Pain Disability Index; **PHQ**, Patient Health Questionnaire; **RA**, Rheumatoid Arthritis; **RMDQ**, Ronald & Morris Disability Questionnaire; **SCID**, Structured Clinical Interview for DSM-IV-TR; **SCL-90-R**, Symptom Checklist 90-R; **SF-36**, Medical Outcomes Survey (short form 36); **SPPB**, Short Physical Performance Battery; **VAS**, Visual Analogue Scale; **15D**, Quality of Life Questionnaire (15 Dimension)

†Prospective studies that measured stigma cross-sectionally; *Study was prospective
